# Radiological findings in the liver of a patient with Rendu-Osler-Weber syndrome

**DOI:** 10.1590/0100-3984.2017.0158

**Published:** 2019

**Authors:** Rafael Amaral Rodrigues, Rodrigo Amaral Rodrigues, Vanessa Carvalho Freitas, Antonio Luis Eiras de Araujo, Daniella Braz Parente

**Affiliations:** 1 Hospital Barra D'Or, Rio de Janeiro, RJ, Brazil.; 2 Universidade Federal do Rio de Janeiro (UFRJ), Rio de Janeiro, RJ, Brazil.; 3 Instituto D'Or de Pesquisa e Ensino (IDOR), Rio de Janeiro, RJ, Brazil.

**Figure 1:**
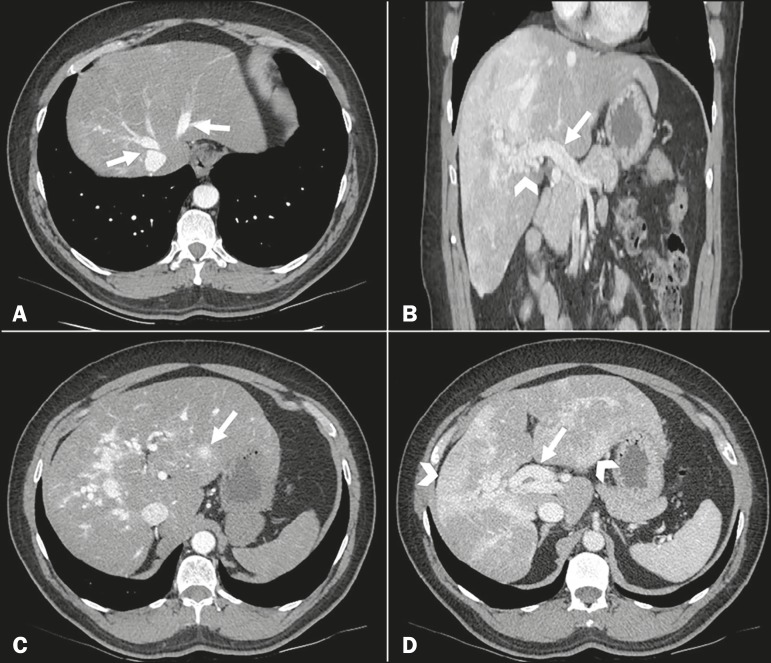
Computed tomography scan of the abdomen in axial slices (**A, C,** and **D**) and in a coronal slice (**B**). **A:** Note the opacification of the hepatic veins in the early arterial phase (arrows). **B:** Heterogeneous opacification of the portal vein during the portal phase (arrow), accompanied by numerous ectatic vascular structures surrounding the hepatic hilum, representing an arteriovenous malformation (arrowhead). **C:** Confluent vascular mass, measuring 1.4 cm, in segment II (arrow). **D:** Extensive areas of altered perfusion in the hepatic parenchyma, in a mosaic pattern (arrow heads), together with increased caliber of the hepatic artery (arrow).

Dear Editor,

A 57-year-old male patient with Rendu-Osler-Weber syndrome presented to the emergency department with a 24-h history of lumbar pain. A computed tomography scan of the abdomen showed liver alterations typical of the syndrome (telangiectasias, shunts, and arteriovenous malformations), which is also known as hereditary hemorrhagic telangiectasia. The examination showed opacification of the hepatic veins in the early arterial phase-a consequence of the arteriovenous shunts (Figure 1A). We observed heterogeneous opacification of the portal vein during the portal phase, with more pronounced enhancement in the intrahepatic branches-a result of portal venous shunt-as well as numerous prominent vessels near the hepatic hilum, corresponding to an arteriovenous malformation (Figure 1B). We also observed a confluent vascular mass, measuring 1.4 cm, located in segment II (Figure 1C). In addition, there were extensive areas of altered perfusion in the hepatic parenchyma, in a mosaic pattern, as well as increased caliber of the hepatic artery at its emergence from the superior mesenteric artery, which was also ectatic (Figure 1D).

Imaging exams have played an important role in the study of liver diseases^(^^1^^-^^5^^)^. Hereditary hemorrhagic telangiectasia is a dominant autosomal disease with a prevalence of 10-20 cases per 100,000 population^(^^6^^)^. It is a rare systemic fibrovascular dysplasia that makes the walls of blood vessels more vulnerable to trauma and spontaneous ruptures^(^^7^^)^. It affects multiple organs and systems, being characterized mainly by the presence of telangiectasias or vascular shunts in the liver, lungs, kidneys, central nervous system, or skin^(^^8^^,^^9^^)^. In adults, it typically manifests as recurrent epistaxis, mucocutaneous telangiectasias, digestive tract hemorrhage, and hemoptysis^(^^9^^,^^10^^)^. Telangiectasias appear gradually, the most common sites being the lips, tongue, palate, fingers, and face. The diagnosis of the syndrome is based on the presence of three of the four diagnostic criteria^(^^8^^)^: mucocutaneous telangiectasias, recurrent spontaneous epistaxis, visceral arteriovenous malformations, and a positive family history.

In Rendu-Osler-Weber syndrome, the liver is the organ most often affected, hepatic involvement being reported in 74% of cases. Hepatic involvement is typically diagnosed 10-20 years after the appearance of the first telangiectasia. In 65% of cases, the liver shows heterogeneous enhancement in the arterial phase, with a mosaic perfusion pattern, which is characterized by areas of altered perfusion, indicative of arterioportal shunts. Hepatic telangiectasias, found in 63% of cases, can be focal or diffuse and are described as rounded lesions, smaller than 10 mm, that are hypervascular in the arterial phase and, in the portal phase, often exhibit density equal to that of the hepatic parenchyma. When such a lesion is larger than 10 mm, as it is in 25% of patients, it is referred to as a confluent vascular mass, comprising areas of grouped multiple telangiectasias or visible shunts^(^^10^^,^^11^^)^.

Vascular shunts, which are seen in 65% of cases of Rendu-Osler-Weber syndrome, appear in one of three forms^(^^11^^)^: arteriovenous (from the hepatic artery to the hepatic vein); arterioportal (from the hepatic artery to the portal vein); and portal-venous (from the portal vein to the hepatic vein). Vascular shunts are associated with complications such as congestive heart failure and portal hypertension^(^^12^^)^. In some cases, there are also hepatic vascular malformations, which can cause a right-to-left shunt, resulting in varying degrees of pulmonary hypertension, heart failure, and hepatic encephalopathy^(^^8^^)^.

The treatment of Rendu-Osler-Weber syndrome includes measures to control epistaxis, as well as surgical removal, radiotherapy, and embolization of vascular malformations, with an emphasis on endovascular treatment^(^^8^^)^.
